# Gold-decorated magnetic nanoparticles design for hyperthermia applications and as a potential platform for their surface-functionalization

**DOI:** 10.1038/s41598-019-40769-2

**Published:** 2019-03-12

**Authors:** L. León Félix, B. Sanz, V. Sebastián, T. E. Torres, M. H. Sousa, J. A. H. Coaquira, M. R. Ibarra, G. F. Goya

**Affiliations:** 1 Laboratory of Magnetic Characterization, Instituto de Física, Universidade de Brasília, Brasília, DF 70910-900 Brazil; 2 Instituto de Nanociencia de Aragón (INA), Universidad de Zaragoza, Zaragoza, 50018 Spain; 3 nB nanoScale Biomagnetics S.L., Zaragoza, Spain; 4 Networking Research Centre on Bioengineering, Biomaterials and Nanomedicine, CIBER-BBN, 28029 Madrid, Spain; 5 Laboratorio de Microscopias Avanzadas (LMA), Universidad de Zaragoza, Zaragoza, 50018 Spain; 6 Green Nanotechnology Group, University of Brasília, Brasília, DF 72220-900 Brazil; 7 Departamento de Física de la Materia Condensada, Universidad de Zaragoza, Zaragoza, 50009 Spain

## Abstract

The integration of noble metal and magnetic nanoparticles with controlled structures that can couple various specific effects to the different nanocomposite in multifunctional nanosystems have been found interesting in the field of medicine. In this work, we show synthesis route to prepare small Au nanoparticles of sizes <d> = 3.9 ± 0.2 nm attached to Fe_3_O_4_ nanoparticle cores (<d> = 49.2 ± 3.5 nm) in aqueous medium for potential application as a nano-heater. Remarkably, the resulted Au decorated PEI-Fe_3_O_4_ (Au@PEI-Fe_3_O_4_) nanoparticles are able to retain bulk magnetic moment M_S_ = 82–84 Am^2^/kg_Fe3O4_, with the Verwey transition observed at T_V_ = 98 K. In addition, the *in vitro* cytotoxicity analysis of the nanosystem microglial BV2 cells showed high viability (>97.5%) to concentrate up to 100 µg/mL in comparison to the control samples. *In vitro* heating experiments on microglial BV2 cells under an ac magnetic field (H_0_ = 23.87 kA/m; *f* = 571 kHz) yielded specific power absorption (SPA) values of SPA = 43 ± 3 and 49 ± 1 μW/cell for PEI-Fe_3_O_4_ and Au@PEI-Fe_3_O_4_ NPs, respectively. These similar intracellular SPA values imply that functionalization of the magnetic particles with Au did not change the heating efficiency, providing at the same time a more flexible platform for multifunctional functionalization.

## Introduction

The fabrication of nanostructured composites allows the possibility of integrating materials with different physical and chemical properties to widen the range of practical applications. Recent attention has been given to the combination of magnetic and metallic nanomaterials in an attempt to profit from a complementary optical and magnetic response^1^. Moreover, the surface of these multifunctional systems can be used as biofunctional platforms to cancer treatment using magnetic hyperthermia that is associated with a heating phenomenon of the magnetic nanoparticles under an alternating magnetic field. This clinical protocol is based on the idea to induce tumor cells death by locally increasing the temperature of ill tissue, when they are previously loaded with magnetic nanoparticles. Other application for multifunctional nanosystems can be found in catalysis, biosensing^2^, magnetic resonance imaging^3,4^, magnetic fluid hyperthermia^5^ and drug delivery^6^.

Pursuing multi-functional uses of a single nanosystem usually requires overcoming incompatible requisites between the final physical and chemical parameters. For a double-purpose hyperthermia nanoparticle (NP), the requirement of strong plasmonic absorption by Au nanoparticles have size and topological constraints that are difficult to match with the best size and shape requirements of Fe_3_O_4_ to maximize the power absorption when exposed to an electromagnetic field. The enhancement in the power absorption must be due to the interfacial exchange interaction between magnetic phases in core–shell systems. It must be noted that Fe_3_O_4_ NPs by themselves have also a photothermal response to a ≈800 nm laser excitation, as demonstrated *in vitro* and *in vivo* by Espinosa *et al*.^5^, although the question of whether the photothermal response of magnetite could be tuned to different optical wavelengths still remains open. Due to these different requirements the synthesis of Au-Fe_3_O_4_ nanoparticles with a core-shell structure has been difficult to achieve in a reproducible way^7–9^. The approach of synthesizing Au-decorated magnetic NPs offers the flexibility to the plasmonic responsiveness by selecting the appropriate size of the Au NPs and its surface functionalization due to the strong adsorption ability^10^ and its reactivity with thiolated^11^ or disulphide groups that makes easier to functionalize them^12,13^. A recent approach to coat polymeric magnetic nanostructures with Au by attaching gold seeds to the NPs surface followed by the reduction of Au has been reported^3^. However, it is somewhat difficult to control the particle aggregation and the uniformity and thickness of the gold shell. Some attempts have been reported about the synthesis of Fe_3_O_4_/Au hybrid structures using different polymers as a platform to attach Au NPs^14,15^, including polymers such as poly(ethylenimine) (PEI), poly(acrylic acid) or dextran. The branched form of PEI is an appealing choice due to the exposed amine groups that provide abundant active sites for chemical modification^16,17^. It also prevents the aggregation driven by the dipolar interaction between magnetic cores, providing better stability to the colloid^11,18,19^ and could provide the advantage of preventing the Fe^2+^ ions to interact with cytoplasmic enzymes promoting the generation of reactive oxygen species through Fenton reaction^20^.

In this work, we reported our initial results of the synthesis and characterization of gold-decorated magnetic nanoparticles to magnetic hyperthermia and as a potential non-toxic carrier for biomedical applications. The reproducibility and morphology of the Au decorated Fe_3_O_4_ NPs was confirmed via high resolution transmission electron microscopy (TEM) and HAADF-STEM images and their magnetic properties are conserved. Subsequently, the Au@PEI-Fe_3_O_4_ NPs were used for hyperthermia on experimental *in vitro*.

## Synthesis of Au@PEI-Fe_3_O_4_ nanoparticles

PEI-coated Fe_3_O_4_ nanoparticles (PEI-Fe_3_O_4_ NPs) were synthesized in one-step using the oxidative hydrolysis method reported elsewhere^21–23^. Subsequently Au nanoparticles were grown on the surface of PEI-Fe_3_O_4_ NPs by a modified method previously reported^11,24^. Briefly, a gold solution was prepared using trisodium citrate (0.068 mmol), sodium borohydride (0.019 mmol) as the reducing agent and gold (III) acetate (0.027 mmol) in 50 mL of deionised water. 40 mL of PEI-Fe_3_O_4_ NPs in a concentration of 0.085 mg/mL was mixed with the Au solution, stirred and heated up to 60 °C for 10 min. Then a solution of 0.1 g trisodium citrate dispersed in 5 mL of deionised water was added. After the formation the Au@PEI-Fe_3_O_4_ NPs, the solution was cooled to room temperature and stirred for 2 hours more. Once the Au was reduced, the solution turned into a deep-red colour indicating the presence of metallic Au NPs and the magnetic separation was an indication of their attachment onto the surface of PEI-Fe_3_O_4_ NPs. The magnetic separation of the Au@PEI-Fe_3_O_4_ NPs were used to wash several times with distilled water until a final pH = 7 was attained.

## Results and Discussion

Our simple procedure to obtain gold-decorated Fe_3_O_4_ NPs in aqueous medium comprises the initial synthesis of PEI-Fe_3_O_4_ NPs through a mild hydrolysis route and subsequent growth of the Au particles onto the magnetic nuclei by the citrate reduction of Au^3+^. The major advantage of this method is the short reaction time and the straightforward growth of Au NPs directly onto the magnetic PEI-Fe_3_O_4_ NPs^11,25^ in aqueous medium^26^. This sequential process allows to independently tune the size and morphology of both Au and Fe_3_O_4_ phases as required by any specific application. The transmission electron microscopy TEM images of as-prepared magnetic nuclei (i.e., before addition of the Au particles) showed an octahedral morphology of the particles, with an amorphous layer of ~2 nm thickness at the surface (see Fig. S1 of the supplementary material), corresponding to the coating of the PEI polymer. Besides stabilizing NPs in colloidal solution, PEI molecules on the surface of magnetic NPs could also act as a reducing agent for the gold ions in solution^27^. Figure S1 also shows the morphology of isolated Au NPs, as obtained by the same synthesis protocol but without adding the PEI-Fe_3_O_4_ NPs.

**Figure 1:**
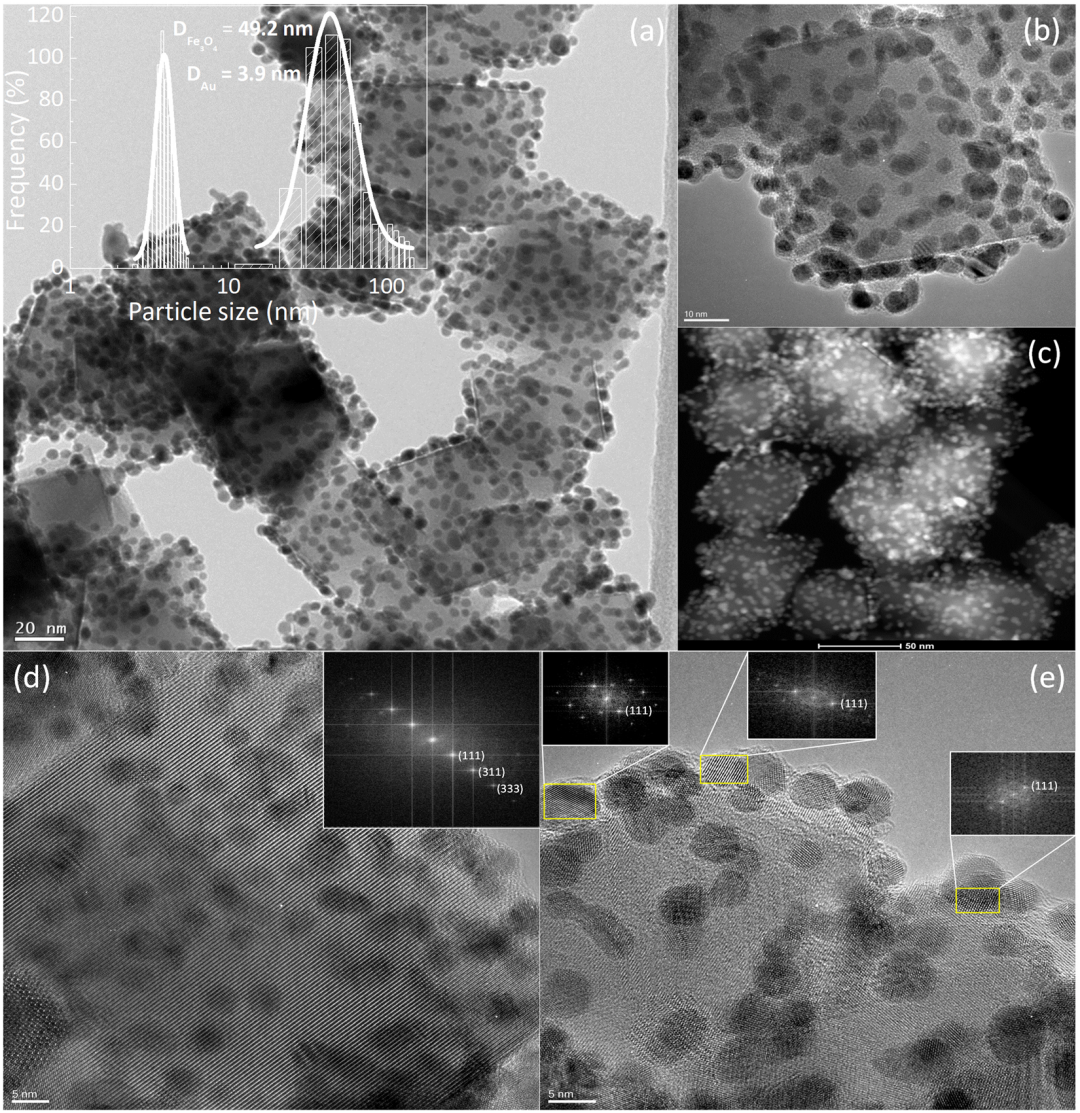
(**a**) TEM image of Au@PEI-Fe_3_O_4_ NPs showing the Au NPs onto the Fe_3_O_4_ surface. Inset: the histogram of particle sizes fitted with a lognormal distribution (solid line), (**b**) detailed view of an individual particle, (**c**) HAADF-STEM image of the Au@PEI-Fe_3_O_4_ NPs, (**d**) and (**e**): HRTEM of a single particle showing the atomic planes. The insets show examples of fast Fourier transform (FFT) spots.

When the two-stage synthesis of Fe_3_O_4_ and Au NPs was performed, the corresponding TEM images for the final Au@PEI-Fe_3_O_4_ system showed that the Au NPs have the same size distribution, and were homogeneously distributed onto the PEI surface (see Fig. 1a,b). The fact that the Au NPs remained attached to the magnetite cores after several washes suggests that they are retained by strong electrostatic interactions of the NH_2_^+^ groups of the PEI^22,28^ and the carboxylic groups of the citrate ions of the Au surface^19,22^. Statistical analysis of TEM images using log-normal functions to fit the size distributions of both phases yielded mean size <d_core_> = 49.2 ± 3.5 for the Fe_3_O_4_ cores, and <d_Au_> = 3.9 ± 0.2 for the Au NPs (see also Fig. S1 of the supporting material). HAADF-STEM images (Fig. 1c) confirmed the homogeneous distribution of the Au NPs (bright dots) within the PEI layer at the Fe_3_O_4_ surface (darker areas)^11^. The crystal lattice planes spacing (Fig. 1d,e) were indexed within the Fd-3m space group correspond to magnetite phase while the patterns from the Au grain locations were fitted using a face-centered cubic structure (space group: Fm-3m). The Fast Fourier Transform (FFT) analysis of the diffraction patterns indicated the crystallographic planes (111), (311) and (333) of the Fe_3_O_4_ phase have interplanar distances of 4.88, 2.53 and 1.63 Å, respectively (inset of Fig. 1d). The corresponding spots from the Au NPs (Fig. 1e) were assigned to the (111) crystallographic plane with interplanar distance of 2.35 Å. These crystal structures were supported by X-ray data (Fig. S2 in the supporting material) through the indexation of the main peaks as the cubic Fe_3_O_4_ spinel phase (JCPDS Card number 75–449) and the FCC phase from the Au NPs (JCPDS Card number 89–3697). The lattice parameters obtained were a = 8.371 Å for Fe_3_O_4_ and a = 4.078 Å for Au, in agreement with values for the corresponding bulk phases^29,30^.

**Figure 2:**
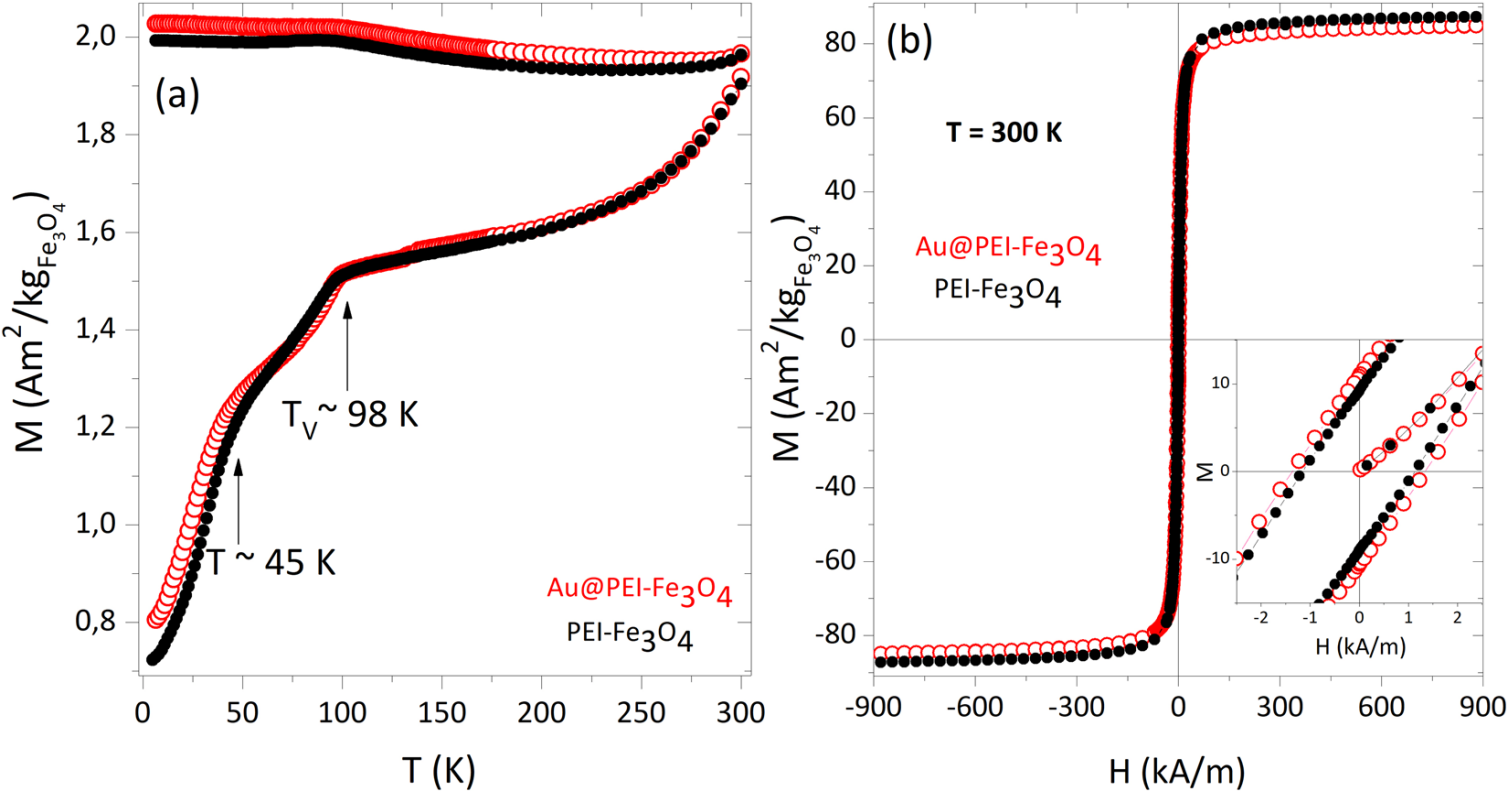
(**a**) DC magnetization curves obtained in zero-field-cooled (ZFC, lower branch) and field-cooled (H_FC_ = 2.39 kA/m, upper branch) modes for PEI-Fe_3_O_4_ (filled black circles) and Au@PEI-Fe_3_O_4_ (open red circles) NPs. (**b**) M vs. H curves at T = 300 K. Inset: magnification of the low-field region of the hysteresis loops.

The magnetic properties of both PEI-Fe_3_O_4_ and Au@PEI-Fe_3_O_4_ NPs were found to be very similar regarding the coercive field (H_c_), saturation magnetization (M_s_) and blocking temperatures (T_B_). This is consistent with the fact that both samples were synthesized from the very same magnetic cores and that no major influence is expected from the non-magnetic Au NPs. The M(T) data measured after zero-field-cooling (ZFC) and field-cooling (FC) protocols (applied field H_FC_ = 2.39 kA/m) are shown in Fig. 2a for PEI-Fe_3_O_4_ and Au@PEI-Fe_3_O_4_ NPs. Both samples showed irreversible behaviour (i.e., separated ZFC and FC branches) up to 300 K, indicating that the magnetic cores are blocked even at room temperature. Consistently, the ZFC curves showed no maximum in temperatures up to 300 K (i.e., the blocking temperature is not below 300 K) as previously reported on similar Fe_3_O_4_ NPs with size ≥ 50 nm^31^. Two distinct ‘shoulders’ were observed in ZFC-mode curves at temperatures T_1_ ≈ 45 K and T_2_ ≈ 98 K. The shoulder at T_2_ has been previously related to the Verwey transition, which occurs at T_V_ = 122 K in bulk Fe_3_O_4_^31–33^. The small bump observed in the FC branch at the same temperature supports this interpretation. The shift of the Verwey transition to lower temperatures has been already reported in Fe_3_O_4_ NPs with sizes smaller than ≈15 nm, and attributed to size^34^ or shape^35^ effects. The origin of the second bump at T_1_ ≈ 45 K is not clear and might be related to thermal relaxation/unblocking processes of the smallest Fe_3_O_4_ (<10 nm) cores observed in TEM images, which is consistent also with the increase of the FC curves at low temperatures due to weak inter-particle interactions of small particles.

Magnetic hysteresis loops of PEI-Fe_3_O_4_ and Au@PEI-Fe_3_O_4_ NPs performed at 300 K (Fig. 2b) showed magnetization saturation values Ms = 82.5 and 84 Am^2^/kg for PEI-Fe_3_O_4_ and Au@PEI-Fe_3_O_4_ NPs, respectively. At 5 K (not shown) the values increased to M_S_ = 90 and 91 Am^2^/kg for PEI-Fe_3_O_4_ and Au@PEI-Fe_3_O_4_ NPs, respectively, essentially those of the bulk Fe_3_O_4_ phase. The coercive fields at 5 K were H_C_ = 33 kA/m for PEI-Fe_3_O_4_ and 29.4 kA/m for Au@PEI-Fe_3_O_4_ NPs, respectively. The coercivity decreased from 5 K to 300 K to small but measurable values of H_C_ = 7.32 and 8.44 kA/m for PEI-Fe_3_O_4_ and Au@PEI-Fe_3_O_4_ NPs, respectively, due to thermal activation approaching the unblocking temperature, which should be therefore close above 300 K. The estimated effective magnetic anisotropy constant ${{\rm{K}}}_{{\rm{eff}}}\,\approx \frac{{\mu }_{0}{{\rm{H}}}_{{\rm{C}}}{{\rm{M}}}_{{\rm{S}}}}{0.96}$ using the low temperature H_C_ and M_S_ values was ${{\rm{K}}}_{{\rm{eff}}}=2.0\times {10}^{4}\,{\rm{J}}/{{\rm{m}}}^{3}$, slightly larger than bulk magnetite (${{\rm{K}}}_{1}=1.1\mbox{--}1.3\times {10}^{4}$ J/m^3^).

**Figure 3:**
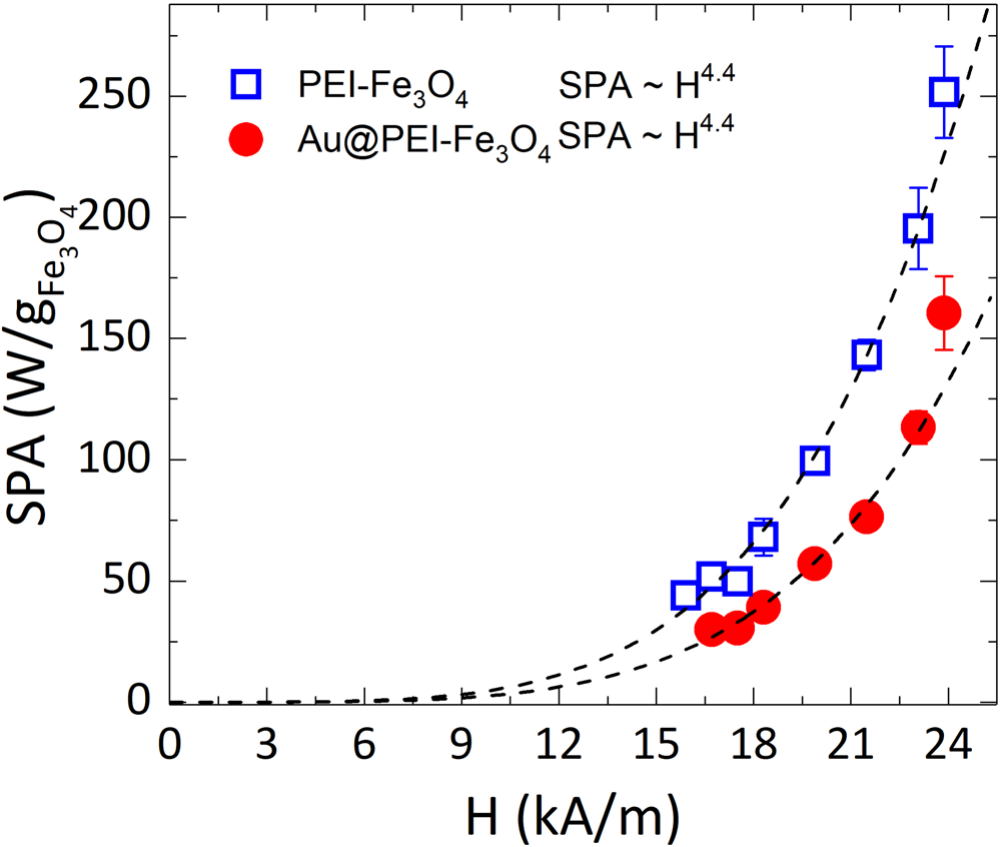
Magnetic field dependence of SPA (*f* = 571 kHz) in *as prepared* nanoparticles suspended in polyvinyl alcohol (PVA). Dotted curves are the fits to experimental data using a power equation: SPA = AH^λ^ (see text).

The physical mechanisms of the power absorption by single domain magnetic nanoparticles under ac magnetic fields have been quite successfully explained by several models for the case of noninteracting particles^36–39^. Assuming a linear response of the magnetization M of a single-domain nanoparticle with volume V_M_ under an ac magnetic field of amplitude H_0_ and frequency ω, the expression

1 $${\bf{P}}={{\boldsymbol{\mu }}}_{0}{\boldsymbol{\pi }}{{\bf{H}}}_{0}^{2}{{\boldsymbol{\chi }}}_{0}\frac{{{\boldsymbol{\omega }}}^{2}{\boldsymbol{\tau }}}{1\,+{({\boldsymbol{\omega }}{\boldsymbol{\tau }})}^{2}}$$

for the power absorption has been given by Rosensweig, where τ is the relaxation time of the magnetic moment, and

2 $${{\rm{\chi }}}_{0}=\frac{{{\rm{M}}}_{{\rm{S}}}}{{{\rm{H}}}_{0}}(\coth \,{\rm{\zeta }}-\frac{1}{{\rm{\zeta }}})$$

is the susceptibility of the magnetic material with ${\rm{\zeta }}=\frac{{{\rm{M}}}_{{\rm{S}}}{{\rm{V}}}_{{\rm{M}}}{{\rm{H}}}_{0}}{{{\rm{k}}}_{{\rm{B}}}{\rm{T}}}$. Therefore, at fixed frequency Eq. (1) reduces to ${\rm{SPA}}={{\rm{AH}}}_{0}^{2}$, where A is a constant that includes all magnetic parameters of the sample. This quadratic dependence given by the LRT is expected to be valid for H_0_ < H_K_, where ${{\rm{H}}}_{{\rm{K}}}=\frac{2{{\rm{M}}}_{{\rm{S}}}}{{{\rm{K}}}_{{\rm{eff}}}}$ is the anisotropy field of the MNPs. This condition is valid working with highly anisotropic particles or very small applied fields. We have performed a systematic investigation of the SPA(H_0_) dependence with applied field at a fixed frequency (at *f* = 571 kHz), using PVA to block the Brownian contribution to the magnetic relaxation and thus mimic the high viscosity at the intracellular medium to compare the results to the *in vitro* measurements (see below). The experimental SPA *vs*. H_0_ data (shown in Fig. 3)were fitted using a phenomenological equation derived from Eq. (1) by assuming all parameters constant except the applied field H_0_ (with H_0_ ≪ H_k_), yielding a power law form

3 $${\bf{S}}{\bf{P}}{\bf{A}}={\bf{A}}{{\bf{H}}}_{0}^{{\boldsymbol{\lambda }}}$$

where λ is an empirical parameter that allows estimating eventual deviations from the LRT regime (i.e. λ = 2)^40,41^. The power absorption of PEI-Fe_3_O_4_ NPs was found to be systematically larger than for Au@PEI-Fe_3_O_4_ NPs at the corresponding applied fields. For H_0_ = 23.9 kA/m the SPA values were 251 ± 18 and 168 ± 15 W/g for PEI-Fe_3_O_4_ and Au@PEI-Fe_3_O_4_ NPs, respectively. Recalling that the magnetic properties of the magnetite nuclei in both samples were essentially the same (that is, the magnetic cores of both samples were from the very same synthesis) and the SPA values are carefully normalized to unit mass of Fe_3_O_4_, the same SPA should be obtained within experimental error. The difference of ≈50 W/g beyond the experimental error bars could be attributed to different agglomeration degrees of both samples, which is consistent to the *in vitro* results discussed below. On the other hand, changes on the Fe_3_O_4_ cores during to process of the incorporation of the Au NPs cannot be discarded, especially partial oxidation of the Fe_3_O_4_ phase yielding some degree of γ-Fe_2_O_3_ (maghemite) phase on the surface and thus changing the magnetic anisotropy of the NPs. Previous work on Fe_3_O_4_@SiO_2_ nanoparticles reported a decrease of the measured SPA with respect to similar but naked Fe_3_O_4_ NPs^42^ but unfortunately the influence of different particle size distributions on the measured SPA cannot be discarded, since no detailed information on the size distributions of the magnetic cores was provided. On the other hand, the work by Bell *et al*.^43^ reported a nearly three-fold increase on the SPA of iron oxide NPs after incorporating Au nanoparticles.

It can be also noticed from Fig. 3 that the fit of the data using Eq. (3) yielded λ ≈ 4.4 ± 0.1 for both Au@PEI-Fe_3_O_4_ and PEI-Fe_3_O_4_ samples, as expected for samples in high viscosity media and having the constituent magnetic cores from the same batch preparation. The similar behaviour regarding magnetic relaxation of the two samples reflects the same average particle sizes and distribution.

**Figure 4:**
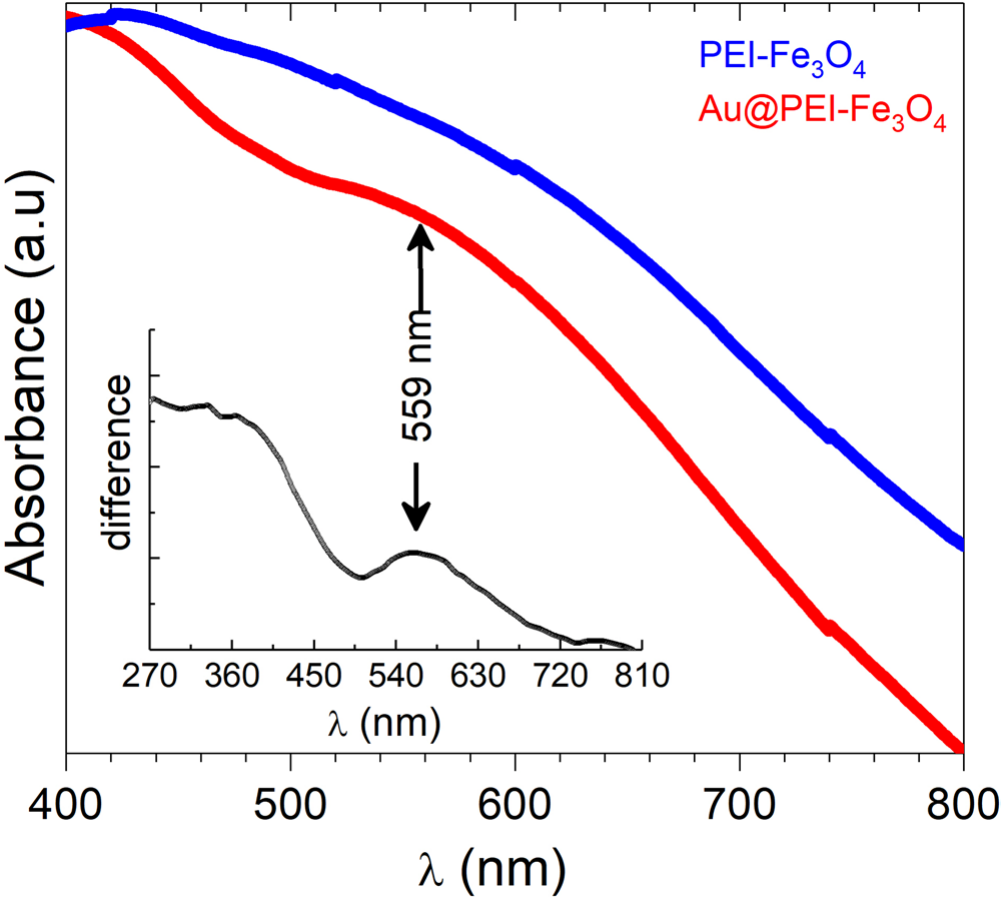
UV-vis spectra of PEI-Fe_3_O_4_ NPs (blue line) and Au@PEI-Fe_3_O_4_ NPs (red line). The inset shows the difference between the two curves and the peak at λ = 559 nm from the Au NPs.

The UV-vis absorption spectra of PEI-Fe_3_O_4_ and Au@PEI-Fe_3_O_4_ NPs dispersed in water exhibit a clear variation of the optical properties (Fig. 4) with the PEI-Fe_3_O_4_ NPs without significant absorbance in the visible region^44^. In contrast, the Au@PEI-Fe_3_O_4_ NPs exhibited a broad absorption band at ≈530 nm that correspond to the absorbance band of the Au NPs^45^. The weak intensity of this broad band is consistent to the small size (<d> = 3.9 nm) of the Au particles produced^46^.

### *In vitro* experiments

A major requirement for the nanosystems to set up a feasible biomedical therapy or protocol is to display low toxicity. To assess the extent of these effects after uptake of MNPs, the toxicity of Au@PEI-Fe_3_O_4_ NPs was evaluated on the microglial cell line (BV2) at different concentrations of NPs from 10 to 100 μg/mL. All experiments were performed after 24 h of NPs co-incubation. High values of cell viability (>97%) were observed for all concentrations of Au@PEI-Fe_3_O_4_ NPs tested (see Fig. S3 in supporting material), consistent with previously reported data^22,28,47^. We mention here that an exception to the above results are connected to those MNPs with some particular NP-coatings (e.g. dextran) that yield to lysosomal incorporation. In these cases, it is well known that iron liberation from NPs and subsequent generation of reactive oxygen species (ROS) within the cell cytoplasm usually result in a significant increase of the cytotoxicity in microglial cells^48^.

**Figure 5:**
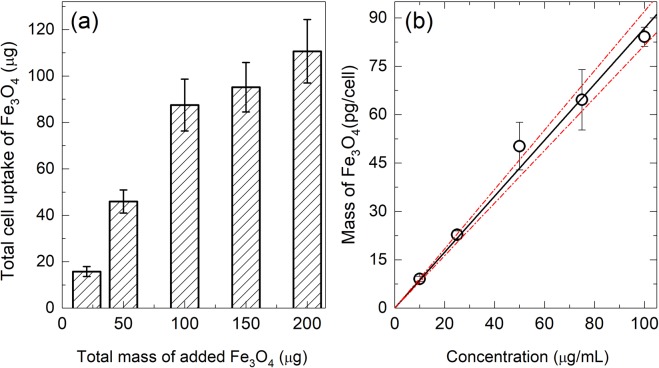
(**a**) Total cellular uptake *vs*. total added amount of Au@PEI-Fe_3_O_4_ NPs for 24 h of incubation time (**b**) Cellular uptake of Au@PEI-Fe_3_O_4_ mass *per* cell *vs*. MNPs concentration, with the best fit to the data (solid line) given by the function $y=(0.868\pm 0.053)\,x$. Dotted lines represent the 95% confidence interval.

A series of quantitative uptake experiments were performed by co-incubating for 24 h with increasing mass of Au@PEI-Fe_3_O_4_ NPs added (from 0 to 200 μg). The results are shown in Fig. 5 indicating a linear trend of the uptake with added mass of NPs. This dependence could be fitted with a linear function $\,y=\,0.868(53)x$, where *y* is the mass of NPs uptaken per cell (in pg) and *x* is the concentration of NPs added in μg/mL. At the highest concentration, the BV2 cells were able to incorporate 87 pg/cell after 24 h incubation, consistent with previously reported data using neuroblastoma cells (SH-SY5Y) incubated with PEI-MNPs^27^.

The surface chemistry of the particles is the main factor influencing cellular uptake, the PEI coating in our particles seems no to hinder the phagocytic activity in BV2 microglial cells, and protein adsorption on the positively charge polymer might be one of the reason for this observed behaviour^49^. The surface charge of the Au@PEI-Fe_3_O_4_ NPs were assessed by zeta potential measurements. The as prepared colloidal PEI-Fe_3_O_4_ NPs in water at pH 7 showed a value of +20.5 mV, as expected for the presence of positively charged amine groups in the polymer backbone. After 24 h of incubation in cell culture medium (complete DMEM), this value changed to −11 mV due to adsorption of proteins onto PEI-Fe_3_O_4_ NPs, in agreement with previous reports^3^. After gold coating, the surface charge of Au@PEI-Fe_3_O_4_ NPs showed a zeta potential of −25 mV in water due to the carboxyl groups of citrate-adsorbed molecules and this value dropped to −12 mV, after the incubation in DMEM cell medium.

**Figure 6:**
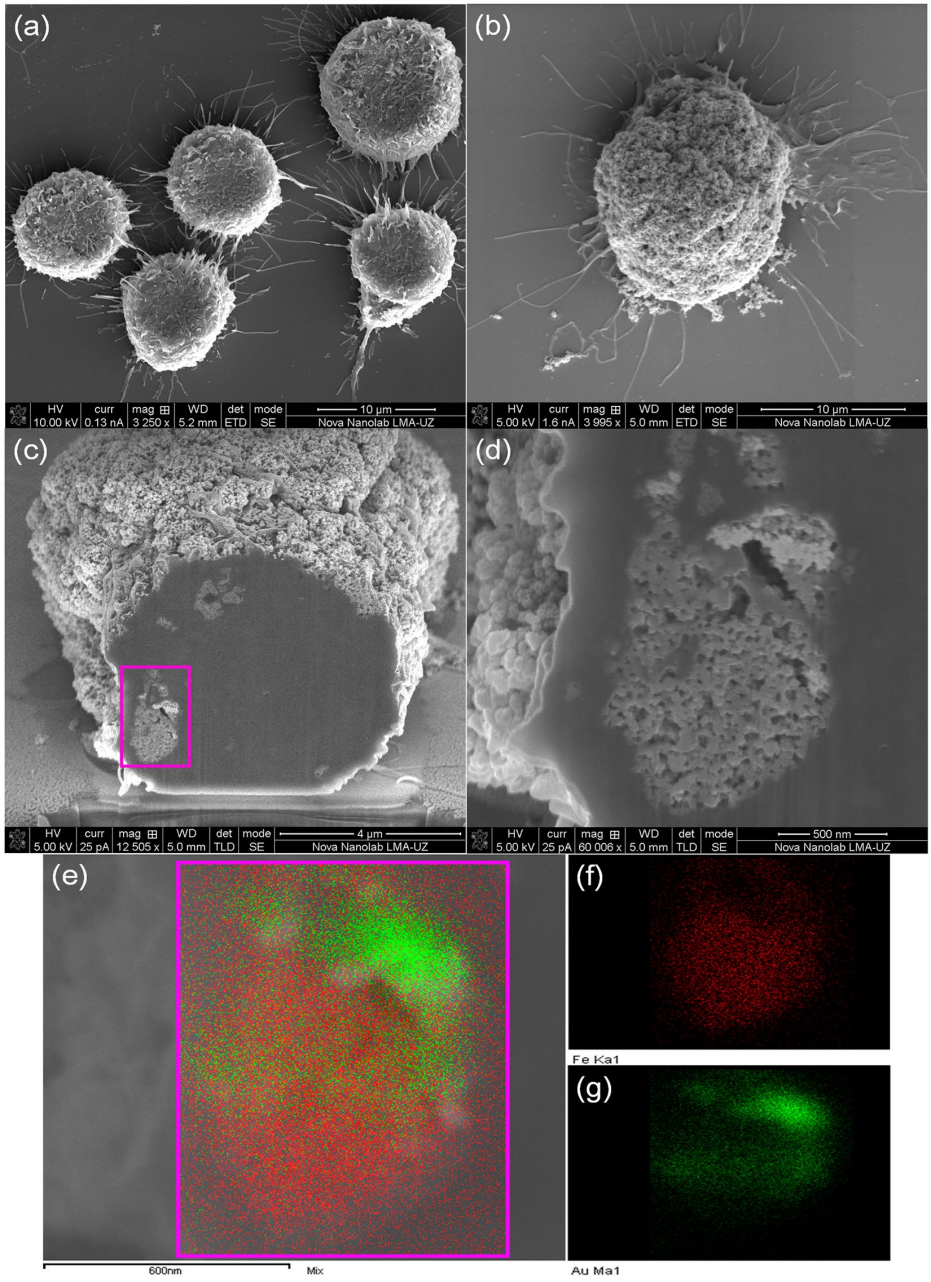
Dual Beam (FIB/SEM) images of (**a**) BV2 control cell, (**b**) a single cell after incubation of BV2 cells with Au@PEI-Fe_3_O_4_ NPs (100 μg/mL) for 24 hours, showing the presence of NPs agglomerates on the cell membrane surface, (**c** and **d**) a cell cross-sectional image confirmed the presence of NPs into cytoplasm; the corresponding EDX mapping images of Fe and Au in the selected area (**e**).

Regarding the final distribution of the particles after incubation, the analysis using FIB-SEM dual beam microscopy showed large amounts of NPs attached to the cell membrane (Fig. 6) for both types of NPs, forming large (~2–5 μm) agglomerates. We did not find any noticeable morphological or adherence changes in the cells before and after incubation with Au@PEI-Fe_3_O_4_ NPs (Fig. 6a). The analysis of the cell cross-sections confirmed the presence of NPs at the intracellular space with the same kind of agglomeration observed at the cell membrane (see Fig. 6c,d). The existences of MNPs were confirmed by EDX spectroscopy through detection of Fe and Au signatures from the cross sections of the intracellular aggregates (see Fig. 6e,g).

**Figure 7:**
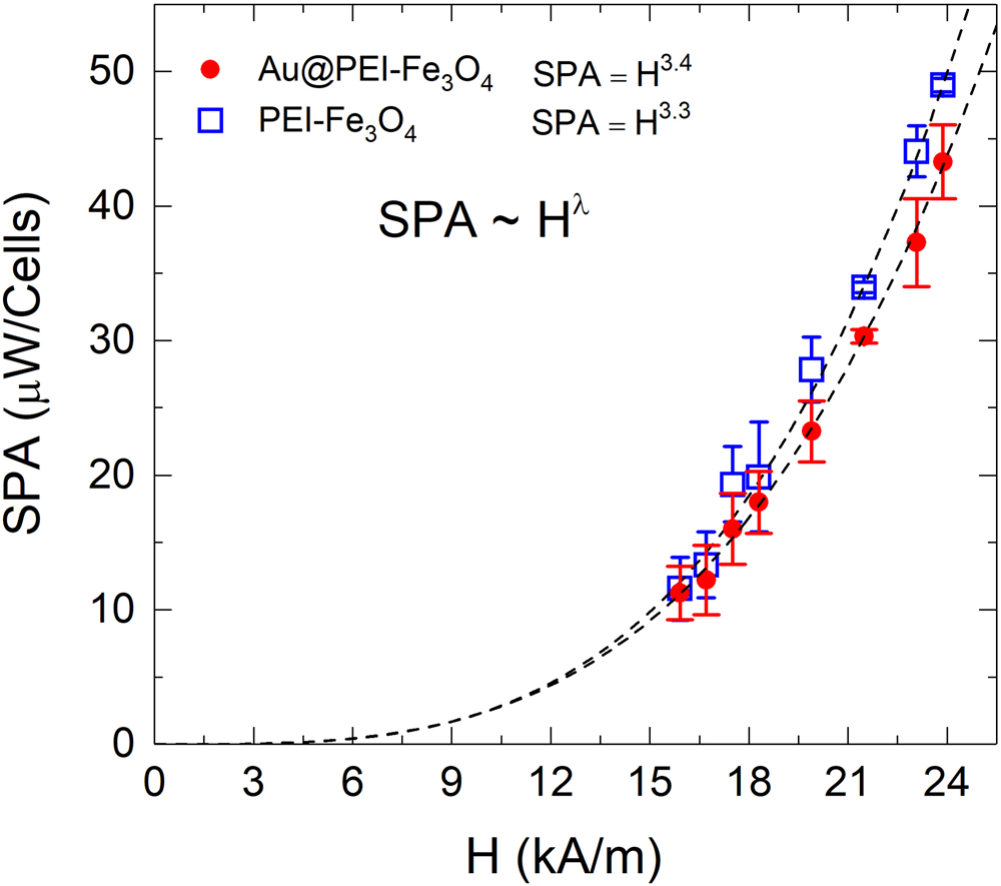
*In vitro* SPA as a function of field amplitude H_0_ (*f* = 571 kHz) for a) PEI-Fe_3_O_4_ NPs (open squares) and Au-PEI-Fe_3_O_4_ (solid circles) nanoparticles within BV2 cells. Dotted lines correspond to the best fit of the data using a power law ${\rm{SPA}}={{\rm{H}}}^{{\rm{\lambda }}}$ (see text).

Figure 7 shows the SPA (H_0_) experimental data (*f* = 571 kHz) for PEI-Fe_3_O_4_ NPs and Au@PEI-Fe_3_O_4_ NPs within the cellular environment (cell pellets containing 9 × 10^6^ cells in a volume of 100 μL). The SPA obtained (H_0_ = 571 kHz; *f* = 24 kA/m, and 100 μg/mL of NPs for cellular uptake) were compared with the SPAs of *as-prepared* colloids (see Fig. 3) and a clear reduction was observed, with SPA = 39.2 and 47.5 μW/cell for Au@PEI-Fe_3_O_4_ and PEI-Fe_3_O_4_ NPs, respectively (see Fig. 7).

The experimental SPA *vs*. H data for both types of NPs were fitted with the same power law in Eq. (3) used for the *as prepared* colloids in water. Similarly to the data from the *as prepared* colloids, the values of the exponent fitted for both samples λ=3.3 ± 0.1 for PEI-Fe_3_O_4_ and λ = 3.4 ± 0.1 for Au@PEI-Fe_3_O_4_ NPs were experimentally coincident as expected from the same magnetic core composition. However, these values were lower than the SPA λ = 4.4 ± 0.1 obtained from the *as prepared* samples dispersed in high viscosity PVA polymer. We attribute the λ > 2 values measured in all cases to the non-linearity of the initial magnetization with H_0_ that precludes the validity of LRT for the present experimental conditions.

We note that for a given set of (*f*, H) parameters, the SPA values measured *in vitro* were systematically lower than for the *as prepared* colloids. Since the inhibition of particle rotation (Brown relaxation) *in vitro* due to the high intracellular viscosity was also present for the PVA-fixed *as prepared* NPs, the lower value from *in vitro* experiments is most likely originated in the dipolar interactions within the NP agglomerates observed from FIB-SEM Dual Beam images. The dipolar interactions within clusters change the magnetic relaxation dynamics^50^. It has been demonstrated by numerical calculations that the dipolar interactions in three-dimensional agglomerates can considerably reduce the SPA in densely-packed clusters. Moreover, in this case the optimal particle size for maximum SPA is shifted towards lower values compared to isolated NPs. It remains to be investigated whether these SPA values can be improved by tuning the particle size and the Au coating for the best use of these NPs as nanoheaters.

## Conclusions

We have obtained Au@PEI-Fe_3_O_4_ NPs by a simple two-step reaction in aqueous medium, with good performance as nanoheaters for magnetic hyperthermia. These particles have very low *in vitro* cytotoxicity, and provided an interesting multifunctional nanoplatform for bimodal application of light and magnetic hyperthermia. It has been demonstrated that the behaviour of SPA with applied field H_0_ is governed only by the properties of the magnetic cores, being experimentally identical for blocked NPs within solid matrix and/or within the intracellular space. However, the natural agglomeration occurring in cells yield dipolar interactions between NPs to decreases the effective SPA and obliges to recalibrate the optimal particle sizes for maximum heat efficiency.

## Methods

### Materials

Au(III) acetate 99.9% was purchased from Alfa Aesar. Sodium borohydride (NaBH_4_) and sodium citrate tribasic di-hydrate (99.99%) were purchased from Sigma Aldrich. Dulbecco’s Modified Eagle Medium (DMEM) containing 10% fetal bovine serum (FBS, Hyclone Lab, InC.), 100 units/mL penicillin, 100 mg/mL streptomycin, and 200 mM L-glutamine were obtained from Sigma-Aldrich. Deionized water was used for all experiments.

### Transmission electron microscopy (TEM)

Average size, distribution and morphology were analysed by transmission electron microscopy (TEM) using a FEI Tecnai T20 microscope, operated at 200 keV. The average particle size (<d>) and size distribution was calculated from histograms after counting N > 500 particles of both Fe_3_O_4_ cores and Au NPs. The data could be fitted with a lognormal distribution. High resolution transmission electron microscopy (HRTEM images were taken using a FEI Tecnai F30 microscope, operated at an acceleration voltage of 300 KV. The microscope was equipped with a HAADF (high angle annular dark field) detector for the STEM mode and EDX (X-ray energy disperse spectrometry) pattern was also studied. Lattice fringes were measured from the fast-Fourier transform of HRTEM images, using Gatan Digital Micrograph. Samples of NPs were prepared by placing one drop of a dilute suspension of NPs in ethanol on a carbon-coated copper grid and evaporating the solvent at room temperature. HRTEM images were used for studied the morphology, grain size and structural information of our samples.

### X-ray diffraction (XRD) measurement

XRD patterns were obtained using a Rigaku Miniflex 600 diffractometer operating at 30 mA and 40 kV from 20 to 80° (2θ value) using Cu K-α radiation (0.15418 nm). The samples were prepared placing a drop of a concentrated NPs suspension on a zero diffraction silicon wafer. Rietveld method analysis was used to confirm the structural analysis of NPs.

### Magnetic measurements

Magnetic properties were determined in dry samples (with nitrogen flow) using a Superconducting Quantum Interference Device (SQUID). Zero-field-cooled (ZFC) and field-cooled (FC) curves were measured between 2 to 300 K, with cooling field H_FC_ = 2.39 kA/m. Magnetization as a function of the field was measured at 5 and 300 K in applied fields up to ± 5570 kA/m. Saturation magnetization (Ms) was obtained by extrapolating to infinite field the experimental results obtained in the high range where magnetization linearly increases with 1/H. Values of the magnetic moment were normalized using the mass of the magnetic core of the Au@PEI-Fe_3_O_4_ NPs. The concentration of Fe and Au was determined by elemental analysis performed using Inductively Coupled Plasma (ICP) technique.

### Specific Power Absorption (SPA) measurement**s**

The parameter to characterize the heating power capacity of our samples is the specific power absorption (SPA), also labelled as specific absorption rate (SAR) or specific loss power (SLP). SPA is described using the expression^51^ ${\rm{SPA}}=\frac{{{\bf{C}}}_{{\bf{L}}{\bf{i}}{\bf{q}}}\,{{\boldsymbol{\delta }}}_{{\bf{L}}{\bf{i}}{\bf{q}}}}{{\rm{\varphi }}}(\frac{{\rm{\Delta }}{\bf{T}}}{{\rm{\Delta }}{\bf{t}}})$, where C_Liq_ and δ_Liq_ are the specific heat capacity and density of the solvent carrier, respectively, ϕ is the mass concentration of the nanoparticles in mg/mL, and ∆T/∆t is the heating rate of the sample during the experiment. In this work the SPA measurements were performed in a commercial magnetic field applicator (nB Nanoscale Biomagnetic S.L., Spain) in a vacuum-insulated Dewar connected to a vacuum pump (10^−7^ mbar) and a fibre optic-based thermometer probe placed at the centre of the sample to determine its temperature. To simulate the high-viscosity conditions of the intracellular medium the nanoparticles were dispersed in a PVA polymeric matrix (10% w/w), resulting in final concentrations of 3.1 mg_Fe3O4_/mL for PEI-Fe_3_O_4_ and 1.66 mg_Fe3O4_/mL for Au@PEI-Fe_3_O_4_ NPs. For *in vitro* experiments both types of NPs (PEI-Fe_3_O_4_ and Au@PEI-Fe_3_O_4_) were incubated with BV2 cells (100 μg/mL) and measurements were performed on cell pellets on an insulated PCR plastic tube, keeping the other parameters unchanged respect to the experiments in *as prepared* colloids (i.e., *f* = 571 kHz and 15.9 ≤ H_0_ ≤ 23.9 kA/m).

### UV-vis spectrophotometry (UV-vis)

UV-vis absorption spectra of the produced nanoparticles were recorded by two spectrophotometers: 1) Thermo Scientific Evolution 220 Diode Array and 2) Jasco (V670). The sample was measured diluted in a 1 mL water solution in a standard quartz cuvette used to quantify the light that is absorbed and scattered by sample. Concentration of Fe in PEI-Fe_3_O_4_ and Au@PEI-Fe_3_O_4_ NPs was determined by UV-vis spectrophotometry (Shymadzu UV-160) using thiocyanate complexation according to the protocol published elsewhere^21,52,53^.

### Zeta potential measurements

The Zeta potential were measured using a Zetasizer Nano ZS90 (Malvern instruments) with a He-Ne laser 633 nm working with a scattering angle of 90°. All samples were measured dispersed on supplemented culture media and room temperature and data were obtained using a monomodal acquisition.

### Cell culture and viability tests

BV2 cells from a murine microglial cell line were cultured in DMEM for *in vitro* studies and maintained at 37 °C 5% CO_2_ and 95% relative humidity. For the cell viability assays, BV2 cells were seeded and incubated into a six-well culture plate (25 × 10^4^ cell/well) for 24 h at 37 °C with 5% CO_2_. The medium was replaced with fresh media containing increasing concentrations of Au@PEI-Fe_3_O_4_ NPs (0, 10, 25, 50, 75 and 100 μg/mL), and incubated overnight. After incubation the medium was removed and the cells were washed twice with PBS. The cells were detached using trypsin and re-suspended in 1 mL of fresh media. Trypan blue was added in equal volume of cell samples. All experiments were conducted in triplicate.

### Cellular uptake test

BV2 cells were planted into six-well plates (25 × 10^4^ cells/well) in a volume of 2 mL. Then the growth media was replaced by medium with increasing amounts of Au@PEI-Fe_3_O_4_ NPs (0, 10, 25, 50, 75 and 100 μg/mL) and incubated for 24 h. The cells were washed with PBS twice times, harvested by trypsinization and suspended in 1 mL of DMEM to count. The pellet precipitated was digested with an acid solution (HCl 6 M and HNO_3_ 65%, 1:1) to quantify the amount of Fe by UV–vis spectrophotometry using the protocol described above.

### Dual Beam (FIB-SEM) analysis

The intracellular distribution of Au@PEI-Fe_3_O_4_ NPs in BV2 cells was studied using a Dual-Beam FIB/SEM analysis (Nova 200 NanoLab, FEI Company) SEM images were taken at 5 and 30 kV with a field emission gun column, and a combined Ga-based 30 kV (10 pA) ion beam to cross-sectioning single cells. This study was complemented by energy-dispersive x-ray spectroscopy (EDX) for chemical analysis. The preparation of the samples was made by seeding BV2 cells on a sterile glass coverslip at a density of 1 × 10^4^ cells/well in 0.5 mL of culture media for 24 hours at 37 °C. After 24 h, the growth medium was replaced with the fresh medium with at a concentration of 100 μg/mL of Au@PEI-Fe_3_O_4_ NPs. After overnight incubation, the cells were washed two times with PBS and fixed with 4% glutaraldehyde solution for 2 hours. After that the coverslips were washed three times with cacodylate buffer (pH 7.2), and then treated with 1% osmium tetroxide and 2.5% potassium ferrocyanate. After being washed, the samples were gradually dehydrated at room temperature via immersion in increasing concentrations of methanol 30% (x2), 50% (x2), 70% (x2), 90% (x2), and 100%. Finally, the samples were coated with gold for FIB-SEM imaging.

## Supplementary information

supporting information
