# Supplementary material for: Gold-decorated magnetic nanoparticles design for hyperthermia applications and as a potential platform for their surface-functionalization
Source: Sci Rep. 2019 Mar 12;9:4185. doi: 10.1038/s41598-019-40769-2 (PMC6414712; doi:10.1038/s41598-019-40769-2)
Supplement: Supplementary file 1 — supporting information [file 41598_2019_40769_MOESM1_ESM.doc]

Supporting Information

Gold-decorated magnetic nanoparticles design for hyperthermia applications and as a potential platform for their surface-functionalization

L. León Félix^a,b,*^, B. Sanz^c^, V. Sebastián^b,d^, T.E. Torres^b,e^, M. H. Sousa^f^, J. A. H. Coaquira^a^, M.R. Ibarra^b,g^ and G. F. Goya^b,g,*^

^a^ Laboratory of Magnetic Characterization, Instituto de Física, Universidade de Brasília, DF 70910-900, Brazil.

^b^ Instituto de Nanociencia de Aragón (INA), Universidad de Zaragoza, 50018 Spain

^c^ nB nanoScale Biomagnetics S.L., Zaragoza, Spain

^d^ Networking Research Centre on Bioengineering, Biomaterials and Nanomedicine, CIBER-BBN, 28029, Madrid, Spain

^e^ Laboratorio de Microscopias Avanzadas (LMA), Universidad de Zaragoza, 50018, Spain.

^f^ Green Nanotechnology Group, University of Brasília, Brasília DF 72220-900, Brazil.

^g^ Departamento de Física de la Materia Condensada, Universidad de Zaragoza, 50009 Spain.

Correspondence and requests for materials should be addressed to L. León Félix ([lizbetlf@gmail.com](mailto:lizbetlf@gmail.com)) and G. F. Goya ([goya@unizar.es](mailto:goya@unizar.es))

**
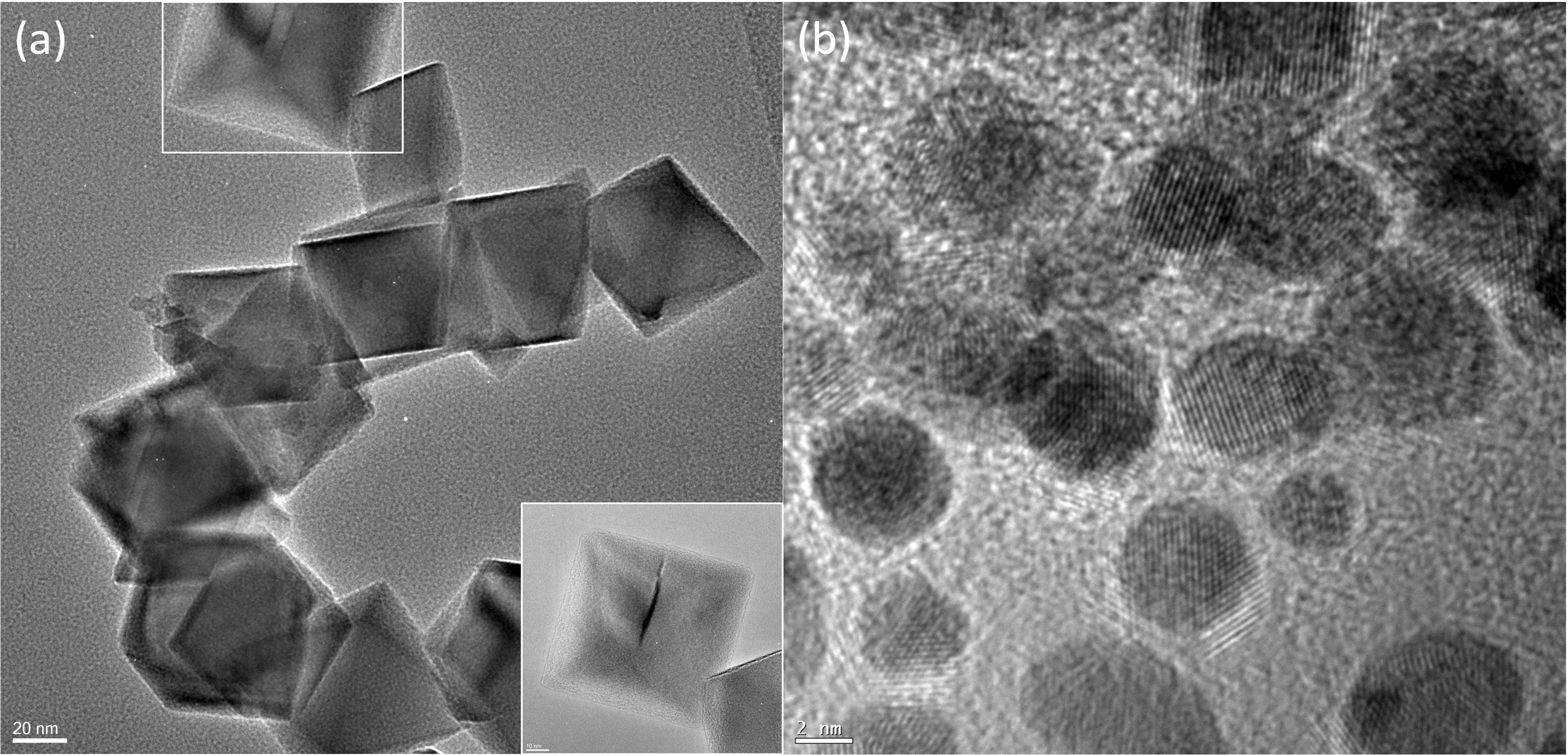
**

**Figure S1.** (a) Magnetite nanoparticles before the gold coated and (b) only pure Au nanoparticles synthesized at the same conditions for magnetite nanoparticles coated.


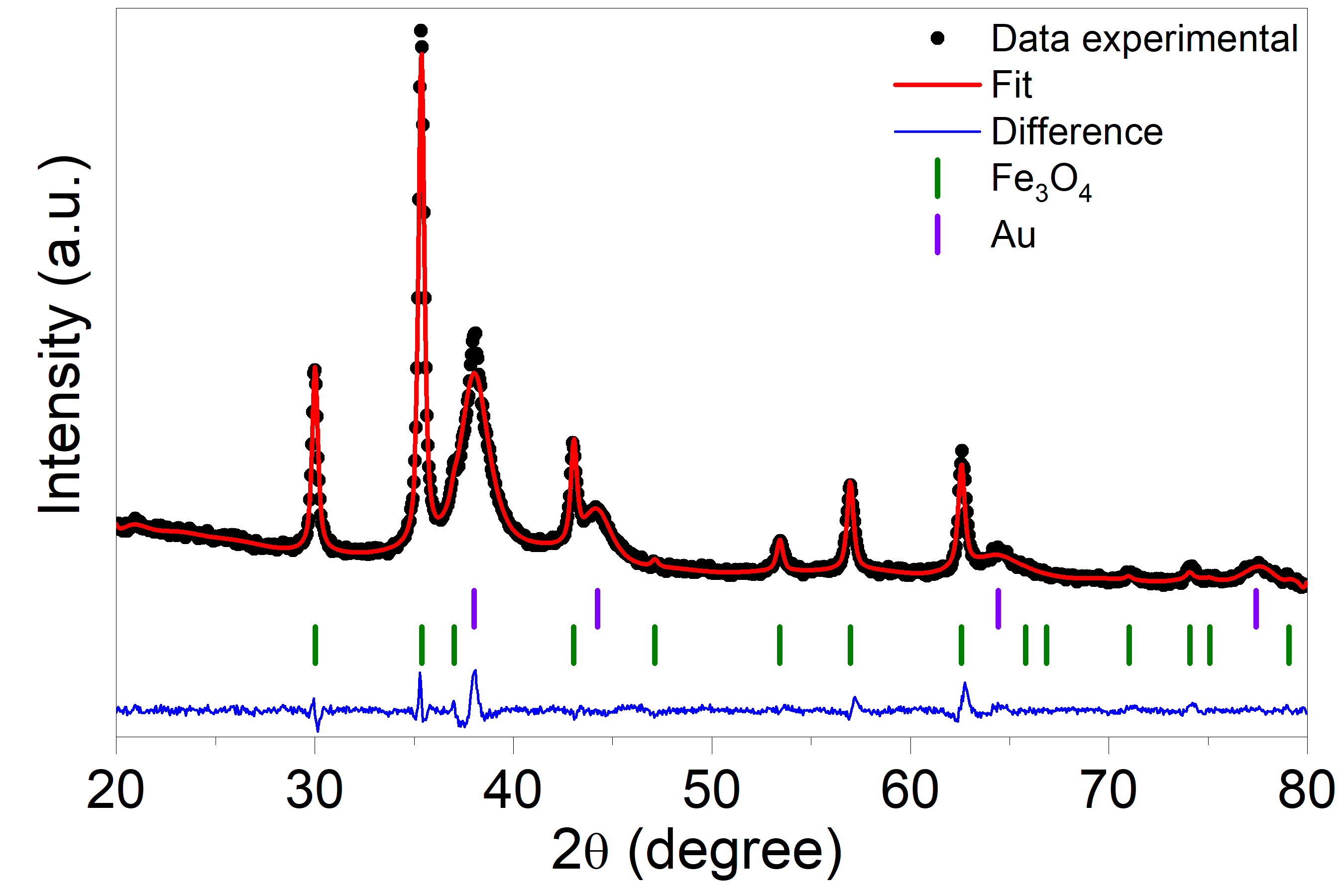


**Figure S2.** X-ray diffraction pattern of Au@PEI-Fe_3_O_4_ NPs. Experimental and calculated data are represented by open circles and red solid line, respectively. The blue line at the bottom represents the difference between experimental and calculated data. The Bragg reflections for each phase are also indicated.


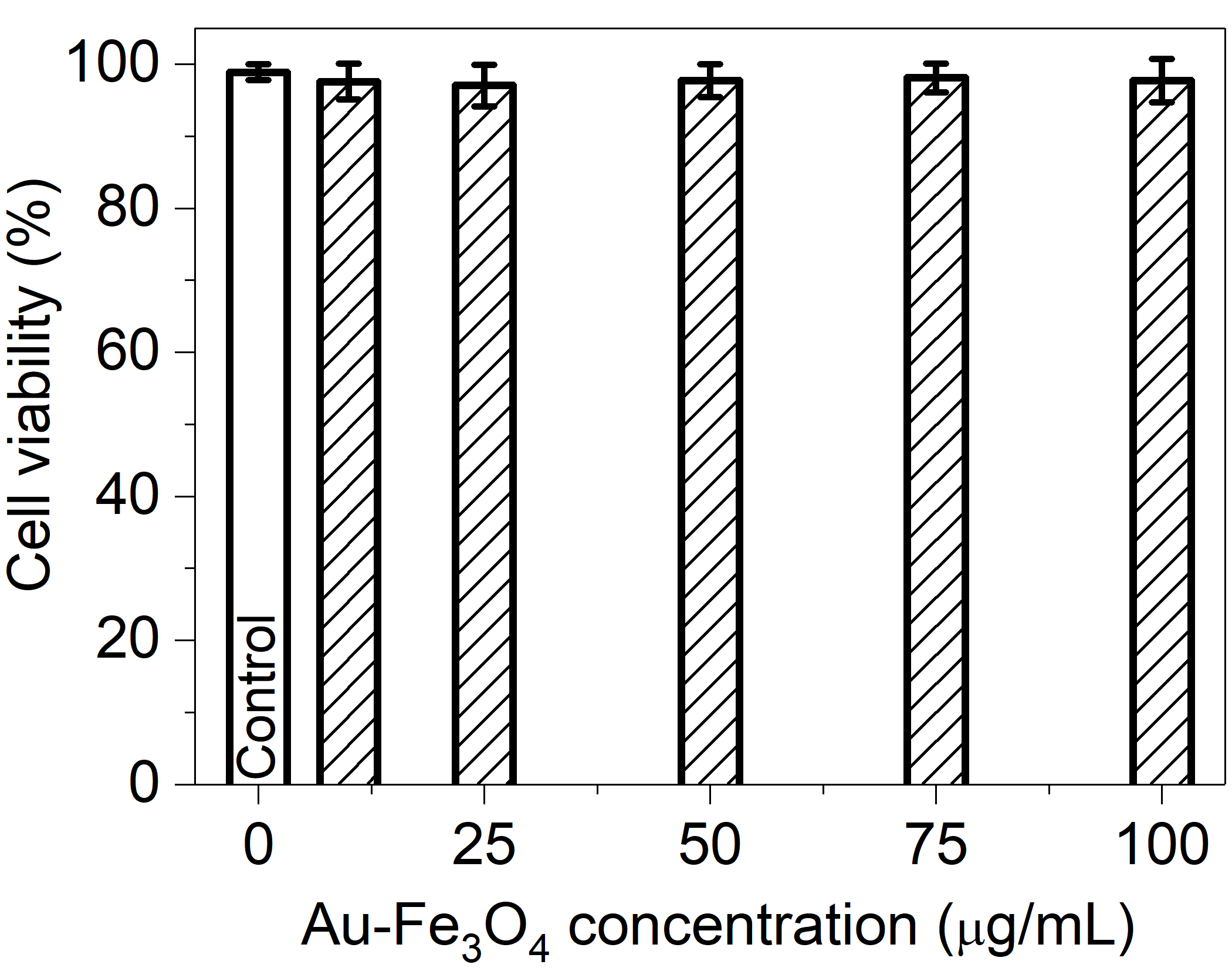


**Figure S3.** Results of cell viability of Au@PEI-Fe_3_O_4_ NPs treated BV2 cells in the presence. Cells incubated with nanoparticles were from 10 to 100 μgmL^-1^ and the incubation time was 24 h. Values of the viability of treated cells were expressed as a percentage of the control cells.
